# Proximal junctional fractures after long-segment instrumented fusion: comparisons between upper instrumented vertebrae and upper instrumented vertebrae + 1

**DOI:** 10.1186/s13018-022-03173-7

**Published:** 2022-05-14

**Authors:** Jen-Chung Liao, Wen-Jer Chen, Shiny Chih-Hsuan Wu

**Affiliations:** Department of Orthopedics Surgery, Bone and Joint Research Center, Chang Gung Memorial Hospital, Chang Gung University, No.5, Fu-Shin Street Kweishian, Taoyuan, 333 Taiwan

## Abstract

**Introduction:**

Proximal junctional failure (PJF) is a well-known complication after long-segment (at least 4 vertebral levels) instrumented fusion. The etiologies of PJF include degenerative processes or are fracture induced. The fracture type of PJF includes vertebral fractures developed at the upper instrumented vertebrae (UIV) or UIV + 1. The purpose of this study was to investigate clinical and radiographic features of these two subtypes of PJF and to analyze risk factors in these patients.

**Method:**

In total, forty-two patients with PJF who underwent revision surgery were included. Twenty patients suffered fractures at the UIV, and the other 22 cases had fractures at UIV + 1. The weighted Charlson Comorbidity Index (CCI) and bone mineral density (BMD) T scores for these patients were recorded. Surgery-related data of index surgery and complications were collected. Radiographic parameters including pelvic tilt (PT), pelvic incidence (PI), sagittal vertical axis (SVA), lumbar lordosis (LL), and PI-LL were recorded in both groups before and after the revision surgery.

**Result:**

Both groups had severe osteoporosis and comorbidities. The interval between the index surgery and revision surgery was shorter in the UIV group than in the UIV + 1 group (8.2 months vs. 35.9 months; p < 0.001). The analysis for radiographic parameters in UIV and UIV + 1 group demonstrated no significant change before and after the revision surgery. However, the preoperative radiographic analysis showed a larger PT (31.5° vs. 23.2°, *p* = 0.013), PI (53.7° vs. 45.3°, *p* = 0.035), and SVA (78.6° vs. 59.4°, *p* = 0.024) in the UIV group compared to the UIV + 1 group. The postoperative radiographic analysis showed a larger PI-LL (27.8° vs. 18.1°, *p* = 0.016) in the UIV group compared to the UIV + 1 group.

**Conclusion:**

PJF in the UIV group tends to occur earlier than in the UIV + 1 group. Moreover, more severe global sagittal imbalances were found in the UIV group than in UIV + 1 group.

## Introduction

Proximal junctional failure (PJF) remains a significant challenge after long instrumented surgeries (defined as fixation of at least four vertebral levels) in adult spinal deformity (ASD). PJF can be caused by adjacent disc degeneration, hardware loosening, and fractures at the upper most instrumented vertebrae (UIV) or in the adjacent vertebrae. [1, 2]. A fracture at the uppermost instrumented vertebrae (UIV) or UIV + 1 after instrumented surgery is considered a fracture type of PJF [3]. The occurrence of fracture type PJF usually leads to revision surgery that often involves simple vertebroplasty (VP)/kyphoplasty (KP) for the fractured vertebrae, or an extension of pedicle instrumentation to provide pain relief and restoration of sagittal balance. In addition to possible perioperative complications, revision surgery by extension of pedicle fixation is also a burden to the patient economically. Revision operations of PJF after long thoracolumbar fusion surgery were reported to be associated with an average cost of 55,000 to 77,000 USD [4, 5]. In contrast, surgical costs of VP or KP are much lower, with reports of an average cost of 15,000 to 27,000 USD [6, 7]. In our experience, revision surgery for PJF that occurs in instrumented fractures of UIV usually requires an extension of pedicle fixation; however, revision surgery with VP/KP is often performed for fractures in UIV + 1. Distinguishing the differences between UIV and UIV + 1 in patients with ASD after long spinal fixation can help surgeons to enhance the quality of care for patients during the follow-up period, caution patients of types of PJF that may be encountered, and allow surgeons to prepare for the treatment of PJF in advance. The purpose of this study was to retrospectively investigate the clinical and radiographic features of these two subtypes of PJF and to analyze the risk factors in these patients.

## Materials and methods

This study was approved by the institutional review board at our hospital. The signed informed consent was waived as this study only involved review of radiographs and medical charts, which did not disclose patient’s personal information. Between January 2005 and December 2019, patients who underwent posterior instrumented fusion for thoracolumbar or lumbar ASD were reviewed. Patients who had undergone revision surgery for symptomatic instrumented fractures at UIV or fractures at UIV + 1 were enrolled. We only included patients who demonstrated degenerative processes in at least 4 vertebral levels and above. Patients who underwent surgeries for infection, inflammatory diseases (for example, ankylosing spondylitis), or tumors were excluded. To reduce statistical bias from different surgical approaches, patients who received combined anterior and posterior surgery to correct their deformities were also excluded. Therefore, we only included patients who underwent surgeries via a posterior approach. All surgeries were performed by one of the two authors. Clinical and radiographic data collection was performed by an independent reviewer who was not involved in the surgical treatment.

**Fig. 1:**
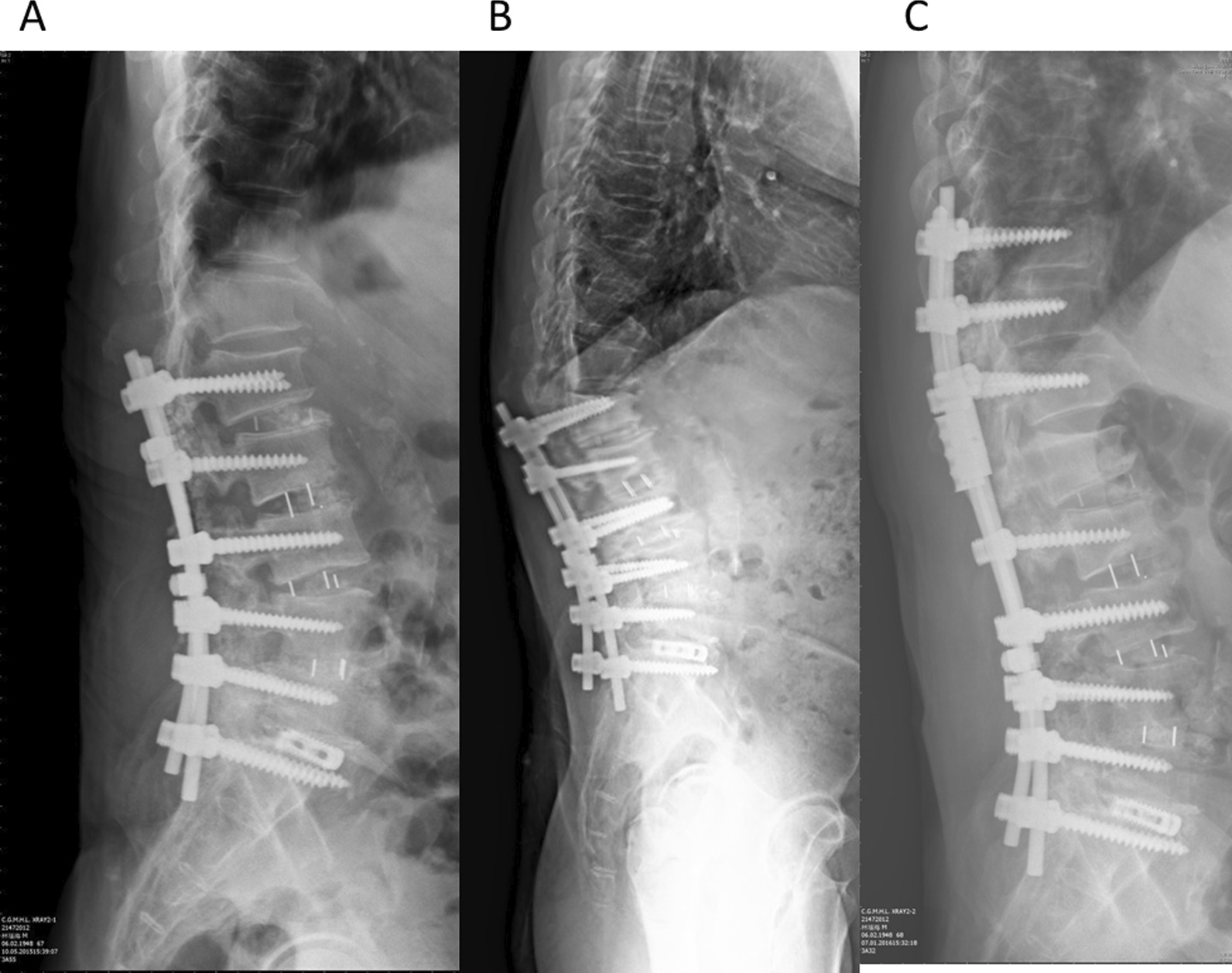
A case in the UIV group: **A** the patient who underwent L1-S1 instrumented fusion, **B** 6 weeks later, suffered from fracture at upper instrumented vertebrae (L1), **C** extended instrumentation to T10 was performed

**Fig. 2:**
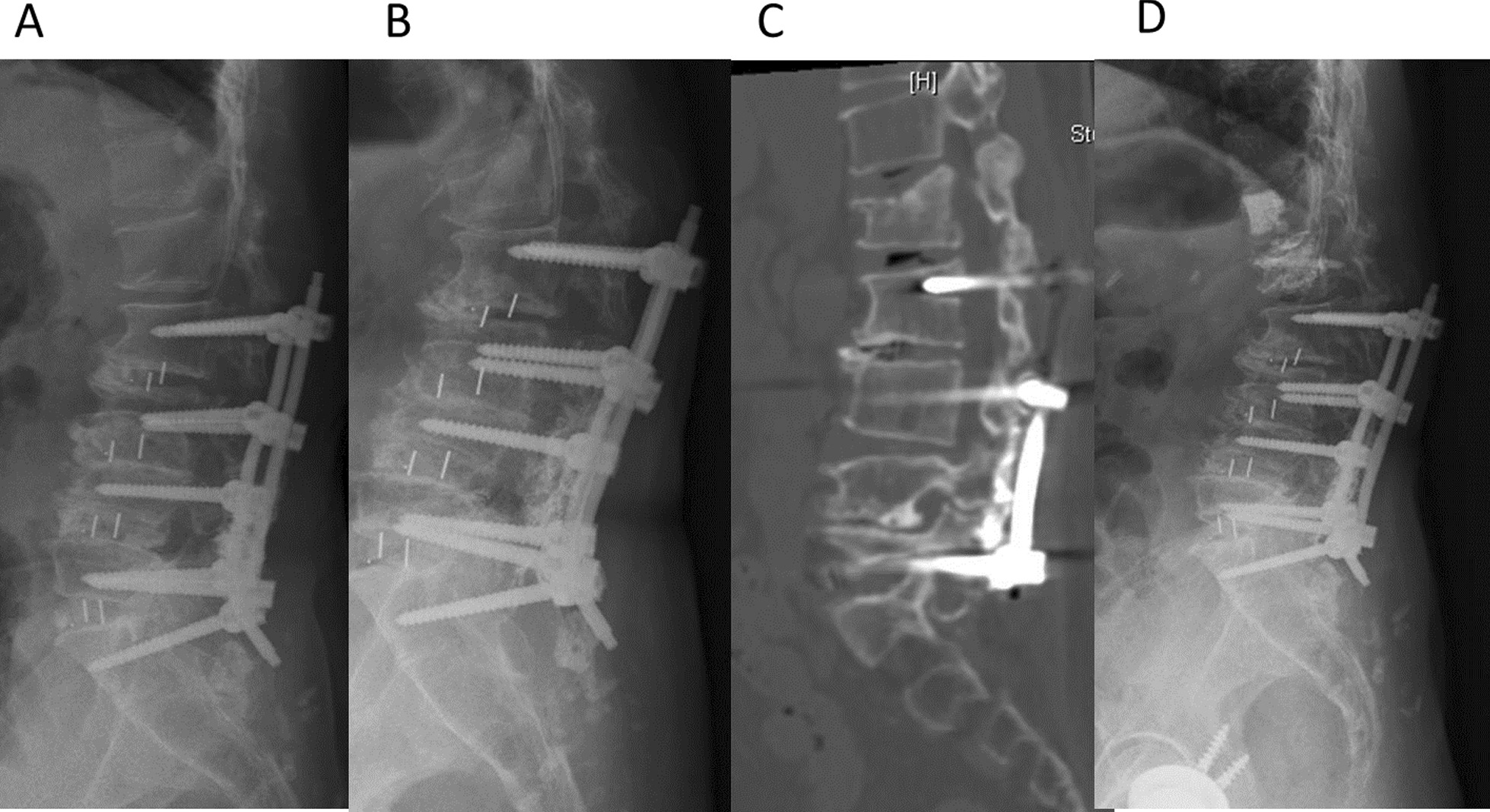
A case in the UIV + 1 group: **A** the patient who underwent L2-S1 instrumented fusion, **B** fracture at upper instrumented vertebrae + 1 (L1) was developed 18 months later, **C** computed tomography showed L1 fracture, **D** L1 vertebroplasty was performed

These enrolled patients were classified into two groups according to the features of the subsequent vertebral fracture: instrumented fracture at the UIV level occurred in 20 patients (UIV group) and adjacent fracture at the UIV + 1 level in 22 patients (UIV + 1 group). The UIV group may demonstrate UIV body collapse only, or UIV body collapse with upward screw penetration into the supra-adjacent disc, or UIV body collapse with supra-adjacent vertebral subluxation. However, the UIV + 1 group demonstrated simple vertebral fracture at UIV + 1 level without destructive change to the UIV and intervertebral disc. Figure 1 shows a case in the UIV group; Fig. 2 demonstrates a case in the UIV + 1 group.

Clinical data collected from medical charts included gender, age at the time of index surgery, body mass index (BMI), the weighted Charlson Comorbidity Index (CCI) [8], the American Society of Anesthesiologists (ASA) physical status classification [9], previous spinal surgery history, pre-existing vertebral fracture history, and the bone mineral density (BMD) T-score. The lowest T-score among values of hip/spine BMD data in each patient was selected for statistical analysis. Surgery-related data of index surgery included instrumented segments, surgical techniques including fusion methods (posterolateral fusion or interbody fusion), whether osteotomy was performed, and the use of S1, iliac screw, or S2-alar-iliac (S2AI) screw. Revision methods for PJF and the interval between index surgery and occurrence of fracture were also collected.

## Radiographic assessment

**Fig. 3:**
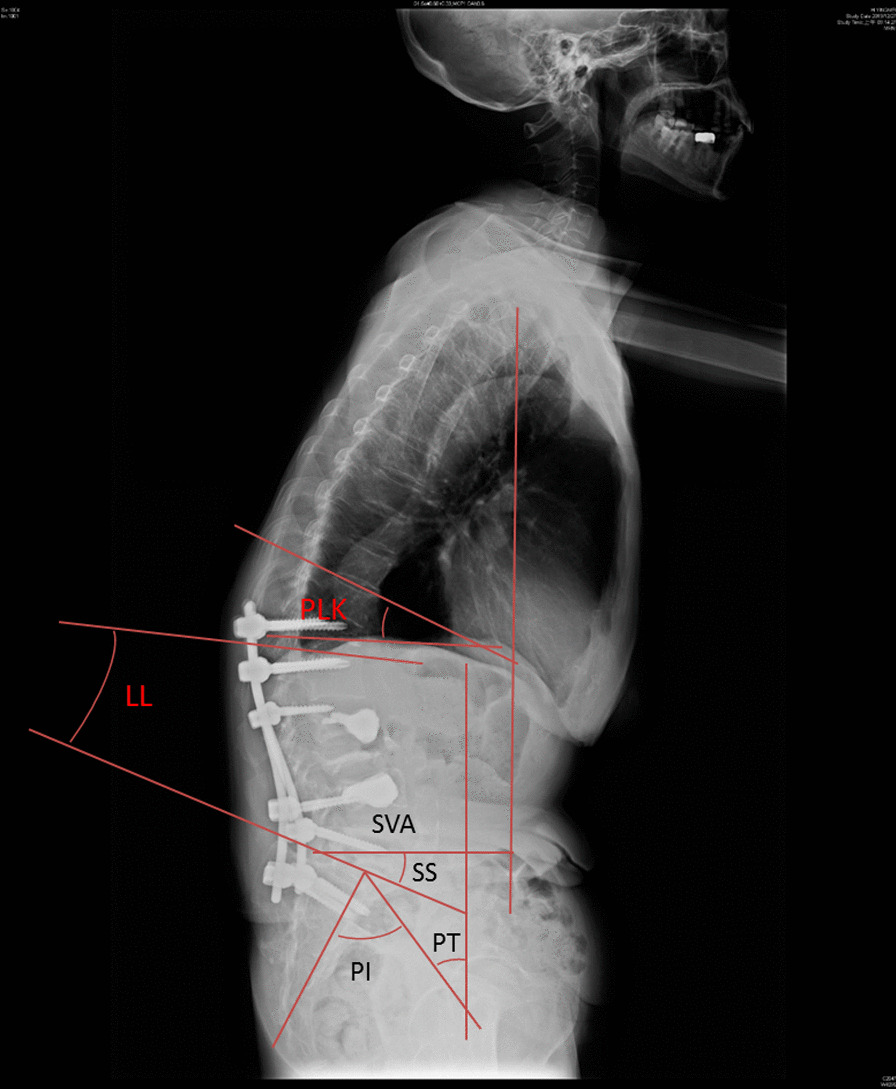
This figure demonstrated radiographic parameters on a whole spine lateral radiograph. *LL* lumbar lordosis, *PI* pelvic incidence, *PLK* proximal local kyphosis, *PT* pelvic tilt, *SS* sacral slope, *SVA* sagittal vertical axis

Lumbar lordosis (LL) angle, sacral slope (SS) angle, pelvic tilt (PT) angle, pelvic incidence (PI) angle, proximal local kyphosis (PLK) angle, coronal scoliosis (CS) angle, and distance (mm) of sagittal vertical axis (SVA) before and after the index surgery were measured. LL angle was measured using the Cobb method of upper endplate of L1-S1; SS angle was measured as the angle between the sacral endplate and a horizontal line; PT angle was measured by the line through the midpoint of the sacral plate and the midpoint of the femoral head axis, and the vertical line; PI angle was measured by the line through midpoint of sacral line and midpoint of the femoral head axis, and the line vertical to sacral plate; PLK angle was measured using the Cobb method of upper endplate of UIV + 1 and lower endplate of UIV presenting in kyphotic angle; CS angle was measured as the maximal scoliosis angle on coronal plain radiographs. The value of SVA was measured as the distance between the C-7 plumb line and the superior posterior corner of the S1 vertebral body in the lateral radiograph. Figure 3 demonstrates how these radiographic parameters were measured. The achievement and importance of a successful harmony of spinopelvic realignment were mentioned by Schwab et al. [10]. The ideal realignment objectives in the sagittal plane included a SVA < 50 mm, PT < 20°, and LL with PI-10° and PI + 10°. A patient with a PT < 20°, LL within PI-10° and PI + 10°, and SVA < 50 mm would get 1 point each. If each of the above criterion was not met, they would receive 0 points. Thus, the total score ranges from 0 to 3 points. The higher the score, the better achievement of spinopelvic harmony.

## Results

There were 42 patients (36 female and 6 male) enrolled in this study, and the average age at the time of the index surgery was 71.1 years. The average number of instrumented vertebrae was 4.9 (range, 4–9). Preoperative diagnosis before the index surgery was degenerative lumbar scoliosis in 17 patients, degenerative lumbar kyphoscoliosis in 5 patients, thoracolumbar kyphoscoliosis in 7 patients, adjacent segment disease of previous instrumented surgery in 13 patients.

### Comparisons of the demographic data between the two groups

The demographic data are listed in Table 1. There were no significant differences in age at the time of the index surgery and revision surgery, sex ratio, BMI, BMD, CCI, and patient’s number of preoperative osteoporosis vertebral fractures (OVFx).

**Table 1:** Patients’ demographic data

Characteristic	UIV group (*N* = 20)	UIV + 1 group (*N* = 22)	*P* values
Age (years)	71.00 ± 5.16	71.09 ± 8.34	0.967
Age (years) (at revision)	71.75 ± 5.22	74.05 ± 8.07	0.286
Gender			
Female	17	19	0.900
Male	3	3	
Fixed vertebrae			
4	5	17	< 0.001
5	6	5	
6	5	0	
7	2	0	
9	2	0	
BMD (T score)	− 3.34 ± 0.97	− 3.26 ± 0.77	0.766
BMI	25.50 ± 3.91	26.07 ± 3.33,	0.618
ASA score	2.87 ± 0.56	2.63 ± 0.64	
CCI	1.60 ± 1.05	1.27 ± 0.83	0.265
Interval (months)	8.18 ± 10.06	35.86 ± 31.10	
*Surgical methods*			
Interbody fusion			
Yes	11	9	0.361
No	9	13	
Corrective osteotomy			
Yes	5	0	0.012
No	15	22	
Sacral or iliac screw			
Yes	12	8	0.002
No	3	19	
OVFx before surgery			
Yes	9	4	0.060
No	11	8	
Fall incidence between index and revision			
Yes	2	13	0.001
No	18	9	
Is index a revision surgery?			
Yes	10	3	0.011
No	10	19	
UIV			
T10	2	0	0.026
T12	5	1	
L1	9	18	
L2	4	3	
LIV			
L4	1	0	0.003
L5	7	19	
S/iliac	12	3	

*BMI* body mass index; *ASA* American Society of Anesthesiologists; *CCI* Charlson Comorbidity Index; *ODI* Oswestry Disability Index; *VAS* visual analogue scale; *OVFx* osteoporosis vertebral fractures

There was a significant difference in the time interval between the index surgery and occurrence of a fracture between the two groups (UIV group: 8.18 ± 10.06 month; UIV + 1 group: 35.86 ± 31.1 months, *P* < 0.001). The UIV group had a significantly higher ratio of patients who underwent corrective osteotomy than the non-revision group (5 vs. 0, *p* = 0.012). The average surgical segments of the index surgery were longer in the UIV group (5.6 vs. 4.2, *p* < 0.001). Furthermore, the UIV group had significantly more patients who underwent instrumentation to the sacrum (12 vs. 8, *p* = 0.002).

## Radiographic data

### Radiographic parameters in the UIV group

Preoperative radiographic parameters including PLK, LL, SS, PT, PI, SVA, and CS were 2.43° ± 7.98°, 20.72 ^o^ ± 16.50°, 22.14 ^o^ ± 11.60°, 31.54° ± 12.98°, 53.68° ± 13.92°, 78.62 mm ± 28.15 mm, and 846° ± 7.80°. Immediately after surgery, these parameters became 2.92° ± 8.99°, 24.34° ± 10.49°, 24.58° ± 10.66°, 28.03° ± 11.55°, 52.16° ± 12.21°, 56.90 mm ± 18.51 mm, and 6.65° ± 6.60°, respectively. Compared to preoperative parameters, only postoperative SAV achieved a statistically significant difference (*p* = 0.006). A preoperative PI-LL was 32.96° ± 17.58°, was corrected to 27.82° ± 12.95° without demonstrating significant difference (*p* = 0.299). However, spinopelvic realignment score achieved a significant change (0.50- > 1.05, *p* = 0.045) with surgery. Table 2 summarizes the radiographic parameters in the UIV group.

**Table 2:** Radiographic Parameters in UIV Group: Preoperative versus Postoperative

Parameter	Preoperative	Postoperative	*P* values
PLK (degree)	2.43 ± 7.98	2.92 ± 8.99	0.856
LL (degree)	20.72 ± 16.50	24.34 ± 10.49	0.412
SS (degree)	22.14 ± 11.60	24.58 ± 10.66	0.493
PT (degree)	31.54 ± 12.98	28.03 ± 11.55	0.371
PI (degree)	53.68 ± 13.92	52.16 ± 12.21	0.716
PI-LL (degree)	32.96 ± 17.58	27.82 ± 12.95	0.299
SVA (mm)	78.62 ± 28.15	56.90 ± 18.51	0.006
CS (degree)	8.46 ± 7.80	6.65 ± 6.60	0.435
Spinopelvic realignment score	0.50 ± 0.76	1..05 ± 1.05	0.065

*PLK* proximal local kyphosis; *LL* lumbar lordosis; *SS* sacral slope; *PT* pelvic tilting; *PI* pelvic incidence; *SVA* sagittal vertical axis; *CS* Cobb’s scoliosis

### *Radiographic parameters in the UIV* + *1 group*

Preoperative radiographic parameters including PLK, LL, SS, PT, PI, SVA, and CS were − 0.91° ± 7.35°, 26.37° ± 16.52°, 22.05° ± 7.66°, 23.27° ± 7.16°, 45.32° ± 10.78°, 59.41° ± 24.88°, and 14.37° ± 16.96°. Immediately after surgery, these parameters were − 1.28° ± 8.19°, 28.73° ± 15.32°, 23.97° ± 7.18°, 22.83° ± 7.97°, 46.80° ± 11.30°, 55.86° ± 17.87°, and 9.04 ± 15.03, respectively. There was no statistically significant difference between preoperative and postoperative data for each radiographic parameter. Preoperative PI-LL was 19.95° ± 11.94°, which was corrected to 18.07° ± 12.23° after surgery without significant improvement (*p* = 0.812). Spinopelvic realignment score also demonstrated no significant change (1.00 preoperative became 1.09 postoperatively, *p* = 0.752) with surgery. Radiographic parameters of the UIV + 1 group are summarized in Table 3.

**Table 3:** Radiographic Parameters in UIV + 1 Group: Preoperative versus Postoperative

Parameter	Preoperative	Postoperative	*P* values
PLK (degree)	− 0.91 ± 7.35	1.28 ± 8.19	0.356
LL (degree)	26.37 ± 16.52	28.73 ± 15.32	0.627
SS (degree)	22.05 ± 7.66	23.97 ± 7.18	0.394
PT (degree)	23.27 ± 7.16	22.83 ± 7.97	0.846
PI (degree)	45.32 ± 10.78	46.80 ± 11.30	0.659
PI-LL (degree)	19.95 ± 11.94	18.07 ± 12.23	0.812
SVA (mm)	59.41 ± 24.88	55.86 ± 17.87	0.590
CS (degree)	14.37 ± 16.96	9.04 ± 15.03	0.276
Spinopelvic realignment score	1.00 ± 0.93	1.09 ± 0.97	0.752

*PLK* proximal local kyphosis; *LL* lumbar lordosis; *SS* sacral slope; *PT* pelvic tilting; *PI* pelvic incidence; *SVA* sagittal vertical axis; *CS* Cobb’s scoliosis

## Comparisons between the UIV and the UIV + 1 group

### Preoperative radiographic parameters

**Fig. 4:**
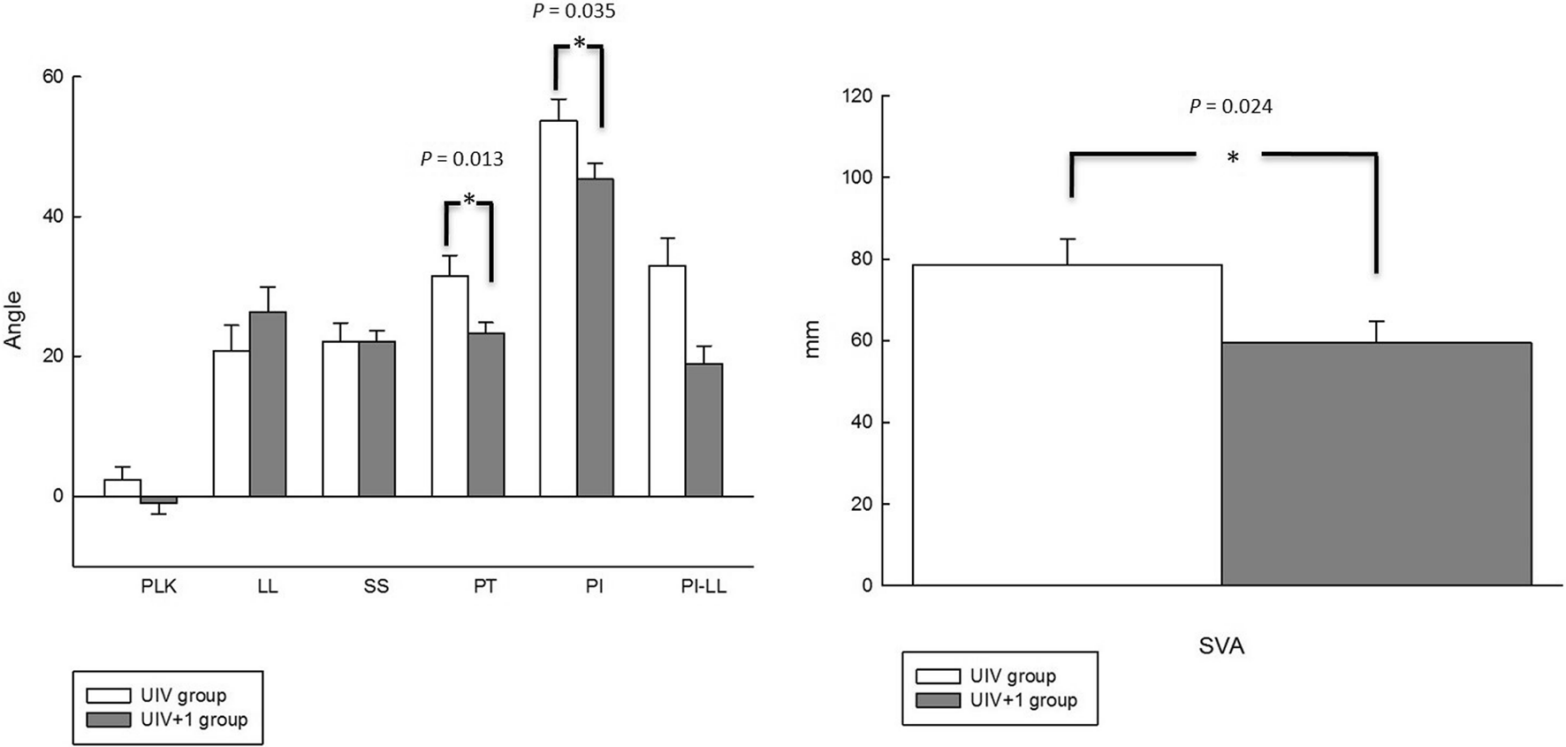
Comparisons of preoperatively radiographic parameters between UIV and UIV + 1 groups. PT, PI, and SVA had significant differences

The UIV group had significantly higher PT (31.54° ± 12.98° vs. 23.27° ± 7.16°, *p* = 0.013), PI (53.68° ± 13.92° vs. 45.32° ± 10.78°, *p* = 0.035), and SVA (78.62° ± 28.15° vs. 59.41° ± 24.88°, *p* = 0.024) than the UIV + 1 group. The average PI-LL was also significantly higher in the UIV group (32.96° ± 17.58° vs. 19.95° ± 11.94°, *p* = 0.004). A lower score for spinopelvic realignment achievement was found in the UIV group preoperatively (0.50 ± 0.76 vs. 1.00 ± 0.93, *p* = 0.065) but without significance. Other preoperative parameters including PLK, LL, SS, CS were not found to be significantly different between the two groups. Figure 4 demonstrates comparisons of preoperatively radiographic data between UIV and UIV + 1 groups.

### Postoperative radiographic parameters

**Fig. 5:**
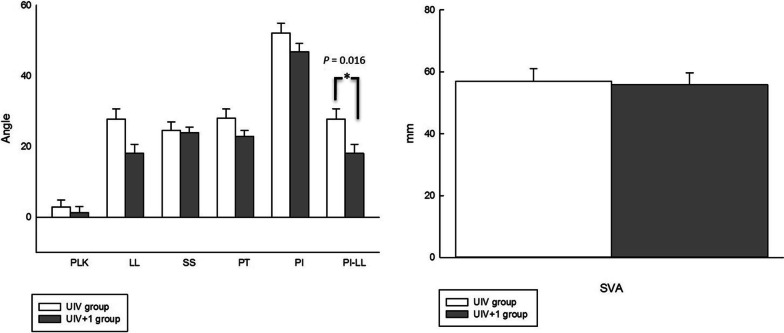
Comparisons of postoperatively radiographic parameters between study and control groups. Only PI-LL had a significant difference

The UIV group had a lower LL (24.34° ± 10.49° vs. 28.73° ± 15.32°, *p* = 0.290), higher PLK (2.92° ± 8.99° vs. 1.28° ± 8.19°, *p* = 0.540), and SVA (56.90° ± 18.51° vs. 55.86° ± 17.87°, *p* = 0.854) than the UIV + 1 group, but did not achieve statistically significant difference. However, both groups had a statistically significant difference for PI-LL (27.82° ± 12.95° vs. 18.07° ± 12.23°, *p* = 0.016). The average postoperative spinopelvic realignment achievement score was 1.05 in the UIV group and 1.09 in the UIV group (*p* = 0.896). No significant differences were found in other postoperative parameters, such as PT, PI, SS, and CS. Figure 5 demonstrates comparisons of the postoperative parameters between the two groups.

## Discussion

Spinal instrumentation and fusion may increase stress on UIV vertebrae and unfused adjacent segments, causing accelerated degeneration or vertebral fracture. There is no consensus for the definition of PJF. Yagi et al. described PJF as symptomatic proximal junctional kyphosis (PJK) necessitating revision surgery [11]. Hostin et al. defined PJF as the presence of high degree symptomatic PJK, or PJK with posterior ligament disruption, PJK due to fractures at UIV or UIV + 1, or by proximal instrument failure [12]. The incidence of PJF after surgery for patients with adult spinal deformities ranges from 2 to 20%; this wide range can be attributed mostly to the heterogeneity of the study populations, surgical methods, and differences in the definition of PJF [13]. We believe that the pathogenesis of PJF due to advanced adjacent disk degeneration or posterior ligament disruption is different to those caused by a fracture. Therefore, we only analyzed patients with the following definition of PJF: UIV and UIV + 1 fractures. We excluded cases with kyphotic change of proximal junction angle caused by adjacent disc degeneration or posterior ligament disruption.

The causes of PJF are multifactorial. Surgical-related factors include excessive sagittal correction, large postoperative pelvic incidence minus lumbar lordosis mismatch, and long spinal fusion including the sacrum and pelvis. Patient-related factors include age > 55 years, high body mass index (BMI), preoperative spinal malalignment, and low bone mineral density (BMD) [3, 14–17]. Our data in both groups showed similar results: average age was over 70 years old, BMD T-score was < − 3.0, and BMI was over 25. In addition, osteoporosis caused by aging, or various comorbidities are apt to occur along with subsequent junctional vertebral fracture after long spinal instrumented fusion [18]. In the current study, CCI in the UIV group was higher than that in the UIV + 1 group (1.65 vs. 1.27), which implies that patients with more comorbidities might be more likely to develop instrumented fractures.

Watanebe et al. were the first authors to describe radiographic and clinical results of proximal junctional vertebral fractures (UIV and UIV + 1) after pedicle screw constructs for spinal deformity [3]. Their experiences demonstrated that proximal instrumented vertebrae collapse usually occurred in the first 6 months after corrective surgery. Similar results were shown in our study where the average surgical interval between index surgery and revision surgery for proximal instrumented vertebrae fracture was 8.2 months, which was significantly shorter than the UIV + 1 group (8.2 vs. 35.9, *P* < 0.001). In addition, they also concluded that marked correction of global sagittal imbalance may be a risk factor for upper instrumented vertebrae fracture. This phenomenon was also shown in our study: SVA correction was 78.6 mm preoperatively to 56.9 mm postoperatively in the UIV group, and 59.4 mm preoperatively to 55.9 mm postoperatively in the UIV + 1 group (*p* < 0.001).

Differences in proximal junctional fractures in either UIV or at UIV + 1 have been demonstrated. Lewis reported their experiences of 13 cases of proximal junctional fractures (7 at UIV and 6 at other proximal adjacent vertebrae) and concluded that a high UIV angle (the sagittal angle of the upper instrumented vertebrae with the horizontal line) on intraoperative lateral radiographs was strongly associated with an UIV fracture [19]. It is noteworthy from their study that a high UIV angle might be related to the performance of osteotomies. Over 70% of patients (5 of 7) in the UIV fracture group received osteotomy procedures, but only 16% of patients (1 of 6) in the proximal adjacent fracture group underwent osteotomy. The same phenomenon was also observed in our study: more segments of osteotomies were performed in the UIV group (5 of 20 in the UIV group vs. 0 of 22 in the UIV + 1 group, *p* = 0.012). We believed that an osteotomy procedure is essential for the correction of global sagittal imbalance. Meanwhile, marked sagittal correction leads to a higher UIV angle, which increases stress on the UIV with subsequent risks of developing UIV fractures particularly in aged, osteoporosis vertebrae. However, there was one point different between Lewis et al. and our study. Our data supported that instrumentation to the sacrum or ilium was more prone to develop UIV fractures instead of UIV + 1 fractures (60% vs. 13%, *p* = 0.003), but Lewis et al. did not support this argument. Sacral or pelvic fixation has been regarded as a risk factor for developing PJF in the literature [17, 20]. However, these studies included all types of PJF such as symptomatic PJK, UIV fracture, and UIV + 1 fracture. They did not distinguish the influence of sacral/pelvic fixation on these subtypes of PJF.

Ha et al. analyzed features of subtypes of acute PJF after correction surgery for thoracolumbar or lumbar deformities. They divided 18 patients with PJF into four subtypes: UIV fracture, UIV + 1 fracture, UIV fracture with junctional subluxation, and fixation failure at UIV [21]. In our series, we separated patients with fracture type PJF into only two subtypes: UIV fracture and UIV + 1 fracture. In fact, cases in the UIV group of our current study included pure UIV, UIV fractures with upper endplate erosion, UIV fractures with mild adjacent segment subluxation, and UIV fractures with backed-out implants. We believed the main reason for revision surgery in these patients described above was due to fracture at the UIV; therefore, we categorized these patients into one group.

Although the main consensus in the literature agreed that UIV and UIV + 1 fractures that occurred after long instrumented fusion were classified as PJF [2, 3, 11, 12, 22], there were similarities and differences in radiographic and clinical features between these two groups. According to the present study, the patients in both groups were osteoporotic as defined by their BMD, had same age and similar BMI/ASA scores/CCI at index surgery. But the average interval between index surgery and revision surgery was statistically longer in the UIV + 1 group (36 months vs. 8 months, *p* < 0.001), which was also observed in Ha et al. and Watanabe et al. [3, 21]. The presence of UIV + 1 fracture usually happens after one fall episode which induces an osteoporotic vertebrae fracture, whereas UIV fractures might be the consequence of direct adjacent stress on the implant and the upper most vertebrae. Preoperative severity of spinal deformity, surgical method of correction, postoperative spinal alignment all contributed to the formation of adjacent stress at the upper most instrument and vertebrae, which leads to the development of an UIV fracture. Therefore, it is meaningful for spine surgeons to clarify UIV fractures and UIV + 1 fractures in PJFs through research.

Based on our data, treatment for osteoporosis was essential for these two groups. Avoiding falling was crucial to prevent the occurrence of UIV + 1 fracture after long instrumented fusion. How to reduce the occurrence of UIV fracture of PJF was more complicated. Some literatures proposed certain techniques to prevent implant-related PJF. Raman et al. suggested that prophylactic vertebroplasty at UIV could be an option to prevent PJF [23]. Viswanathan et al. proposed a hybrid method by combining a pedicle screw-rod construct and sub-laminar banding to prevent proximal junctional stress [24]. Rodriguez-Fontan et al. reported an easier method which used Mersilene tape to stabilize the spinal process between the UIV and UIV + 1 or 2 to prevent PJF [25]. We thought that more clinical studies are required to approve efficacy of these techniques.

Finally, this study has several limitations. First, there may still be case selection bias because this was a small retrospective study not a randomized controlled trial. Given that the entire cohort comprised mostly of women, our findings cannot validly be applied to all patients undergoing ASD surgery. The second limitation is the limited case number included in this study. There were some subtypes in the UIV group, if stratifying patients into more groups would result in smaller samples and difficulty in statistical analysis. Therefore, large cohort studies may be required in the future to validate the results of this study.

## Conclusion

A lower T-score was seen in both UIV and UIV + 1 groups. PJF in the UIV group tends to occur earlier than the UIV + 1 group. Moreover, more severe global sagittal imbalance was found in the UIV group than in the UIV + 1 group. Correction by osteotomy for sagittal imbalance and fixation to sacrum or pelvis were risk factors to develop PJF with UIV fracture; prophylactic procedures at proximal junctional segments might be required to reduce possibility of UIV fracture.

## Data Availability

The data used to support the finding of this study are available from the corresponding author upon request.

## Abbreviations

PJF: Proximal junctional failure
UIV: Upper instrumented vertebrae
CCI: Charlson Comorbidity Index (CCI)
BMD: Bone mineral density
PT: Pelvic tilt
PI: Pelvic incidence
SVA: Sagittal vertical axis
LL: Lumbar lordosis
VP: Vertebroplasty
KP: Kyphoplasty
ASD: Adult spinal deformity
ASA: American Society of Anesthesiologists

## References

[1] Bridwell KH, Lenke LG, Cho SK, Pahys JM, Zebala LP, Dorward IG, Cho W, Baldus C, Hill BW, Kang MM (2013). Proximal junctional kyphosis in primary adult deformity surgery: evaluation of 20 degrees as a critical angle. Neurosurgery.

[2] Smith MW, Annis P, Lawrence BD, Daubs MD, Brodke DS (2015). Acute proximal junctional failure in patients with preoperative sagittal imbalance. Spine J.

[3] Watanabe K, Lenke LG, Bridwell KH, Kim YJ, Koester L, Hensley M (2010). Proximal junctional vertebral fracture in adults after spinal deformity surgery using pedicle screw constructs: analysis of morphological features. Spine.

[4] Hart RA, Prendergast MA, Roberts WG, Nesbit GM, Barnwell SL (2008). Proximal junctional acute collapse cranial to multi-level lumbar fusion: a cost analysis of prophylactic vertebral augmentation. Spine J.

[5] Theologis AA, Miller L, Callahan M, Lau D, Zygourakis C, Scheer JK, Burch S, Pekmezci M, Chou D, Tay B, Mummaneni P, Berven S, Deviren V, Ames CP (2016). Economic impact of revision surgery for proximal junctional failure after adult spinal deformity surgery: a cost analysis of 57 operations in a 10-year experience at a major deformity center. Spine.

[6] Ong KL, Lau E, Kemner JE, Kurtz SM (2013). Two-year cost comparison of vertebroplasty and kyphoplasty for the treatment of vertebral compression fractures: are initial surgical costs misleading?. Osteoporos Int.

[7] Alvi MA, Zreik J, Yolcu YU, Goyal A, Kim DK, Kallmes DF, Freedman BA, Bydon M (2020). Comparison of costs and postoperative outcomes between vertebroplasty and kyphoplasty for osteoporotic vertebral compression fractures: analysis from a state-level outpatient database. World Neurosurg.

[8] Charlson ME, Pompei P, Ales KL, MacKenzie CR (1987). A new method of classifying prognostic comorbidity in longitudinal studies: development and validation. J Chronic Dis.

[9] Sankar A, Johnson SR, Beattie WS, Tait G, Wijeysundera N (2014). Reliability of the American society of anesthesiologists physical status scale in clinical practice. Br J Anaesth.

[10] Schwab F, Patel A, Ungar B, Farcy JP, Lafage V (2010). Adult spinal deformity-postoperative standing imbalance: how much can you tolerate? An overview of keyparameters in assessing alignment and planning corrective surgery. Spine.

[11] Yagi M, Rahm M, Gaines R, Maziad A, Ross T, Kim HJ, Kebaish K, Boachie-Adjei O (2014). Complex Spine Study Group. Characterization and surgical outcomes of proximal junctional failure in surgically treated patients with adult spinal deformity. Spine.

[12] Hart R, McCarthy I (2013). International spine study group. Identification of decision criteria for revision surgery among patients with proximal junctional failure after surgical treatment of spinal deformity. Spine.

[13] Yagi M, Fujita N, Okada E, Tsuji O, Nagoshi N, Asazuma T, Ishii K, Nakamura M, Matsumoto M, Watanabe K (2018). Fine-tuning the predictive model for proximal junctional failure in surgically treated patients with adult spinal deformity. Spine.

[14] Kim YJ, Bridwell KH, Lenke LG, Rhim S, Cheh G (2006). Sagittal thoracic decompensation following long adult lumbar spinal instrumentation and fusion to L5 or S1: causes, prevalence, and risk factor analysis. Spine.

[15] Le Huec JC, Faundez A, Dominguez D, Hoffmeyer P, Aunoble S (2015). Evidence showing the relationship between sagittal balance and clinical outcomes in surgical treatment of degenerative spinal diseases: a literature review. Int Orthop.

[16] Nguyen NL, Kong CY, Hart RA (2016). Proximal junctional kyphosis and failure-diagnosis, prevention, and treatment. Curr Rev Musculoskelet Med.

[17] Liao JC, Chen WJ (2022). The influences of spinopelvic parameters and risk factors on development of proximal instrumented fracture after posterior instrumentation. World Neurosurg.

[18] Nakahashi M, Uei H, Tokuhashi Y, Maseda M, Sawada H, Soma H, Miyakata H (2019). Vertebral fracture in elderly female patients after posterior fusion with pedicle screw fixation for degenerative lumbar pathology: a retrospective cohort study. BMC Musculoskelet Disord.

[19] Lewis SJ, Abbas H, Chua S, Bacon S, Bronstein Y, Goldstein S, Magana S, Sullivan K, Dold AP, Bodrogi A (2012). Upper instrumented vertebral fractures in long lumbar fusions: what are the associated risk factors?. Spine.

[20] O’Shaughnessy BA, Lenke LG, Bridwell KH, Cho W, Zebala LP, Chang MS, Auerbach JD, Crawford CH, Koester LA (2012). Should symptomatic iliac screws be electively removed in adult spinal deformity patients fused to the sacrum?. Spine.

[21] Ha KY, Kim YH, Oh IS, Seo JY, Chang DG, Park HY, Min HK, Kim SI (2019). Clinical and radiographic features of subtypes of acute proximal junctional failures following correction surgery for degenerative sagittal imbalance. World Neurosurg.

[22] Park SJ, Lee CS, Park JS, Lee KJ (2020). Should thoracolumbar junction be always avoided as upper instrumented vertebra in long instrumented fusion for adult spinal deformity?: risk factor analysis for proximal junctional failure. Spine.

[23] Raman T, Miller E, Martin CT, Kebaish KM (2017). The effect of prophylactic vertebroplasty on the incidence of proximal junctional kyphosis and proximal junctional failure following posterior spinal fusion in adult spinal deformity: a 5-year follow-up study. Spine J.

[24] Viswanathan VK, Ganguly R, Minnema AJ, DeVries Watson NA, Grosland NM, Fredericks DC, Grossbach AJ, Viljoen SV, Farhadi HF (2018). Biomechanical assessment of proximal junctional semi-rigid fixation in long-segment thoracolumbar constructs. J Neurosurg Spine.

[25] Rodriguez-Fontan F, Reeves BJ, Noshchenko A, Ou-Yang D, Kleck CJ, Cain C, Burger-Van der Walt E, Patel VV (2020). Strap stabilization for proximal junctional kyphosis prevention in instrumented posterior spinal fusion. Eur Spine J.

